# Acetylcholinesterase immobilization and characterization, and comparison of the activity of the porous silicon-immobilized enzyme with its free counterpart

**DOI:** 10.1042/BSR20150154

**Published:** 2016-03-16

**Authors:** Muhammad Saleem, Muhammad Rafiq, Sung-Yum Seo, Ki Hwan Lee

**Affiliations:** * Department of Chemistry, Kongju National University, Gongju, Chungnam 314-701, Republic of Korea; † Department of Biology, Kongju National University, Gongju, Chungnam 314-701, Republic of Korea; ‡ Department of Biochemistry and Biotechnology, the Islamia University of Bahawalpur, Pakistan

**Keywords:** acetylcholinesterase, biocatalyst, hydrolysis, immobilization, mesoporous, physical adsorption

## Abstract

A successful prescription is presented for acetylcholinesterase physically adsorbed on to a mesoporous silicon surface, with a promising hydrolytic response towards acetylthiocholine iodide. The catalytic behaviour of the immobilized enzyme was assessed by spectrophotometric bioassay using neostigmine methyl sulfate as a standard acetycholinesterase inhibitor. The surface modification was studied through field emission SEM, Fourier transform IR spectroscopy, energy-dispersive X-ray spectroscopy, cathode luminescence and X-ray photoelectron spectroscopy analysis, photoluminescence measurement and spectrophotometric bioassay. The porous silicon-immobilized enzyme not only yielded greater enzyme stability, but also significantly improved the native photoluminescence at room temperature of the bare porous silicon architecture. The results indicated the promising catalytic behaviour of immobilized enzyme compared with that of its free counterpart, with a greater stability, and that it aided reusability and easy separation from the reaction mixture. The porous silicon-immobilized enzyme was found to retain 50% of its activity, promising thermal stability up to 90°C, reusability for up to three cycles, pH stability over a broad pH of 4–9 and a shelf-life of 44 days, with an optimal hydrolytic response towards acetylthiocholine iodide at variable drug concentrations. On the basis of these findings, it was believed that the porous silicon-immobilized enzyme could be exploited as a reusable biocatalyst and for screening of acetylcholinesterase inhibitors from crude plant extracts and synthesized organic compounds. Moreover, the immobilized enzyme could offer a great deal as a viable biocatalyst in bioprocessing for the chemical and pharmaceutical industries, and bioremediation to enhance productivity and robustness.

## Abstract (precis)

The physically adsorbed acetylcholinesterase on mesoporous silicon surface is presented. The catalytic behavior of immobilized enzyme was assessed by spectrophotometric bioassay. The immobilization enhanced the reusability, shelf life and thermal as well as pH stability

## INTRODUCTION

Electrochemical etching of single crystalline silicon in hydrofluoric acid (HF)-based electrolytic solutions leads to the formation of various pore arrays, known as porous silicon [1]. The pores are generated by means of anodic electrochemical or photoelectrochemical etching of a silicon wafer under galvanostatic conditions. Porous silicon material with different optical features can be produced by varying the etching parameters, including etching time and anodization current density [2]. The unique features of porous silicon make this material a frontline candidate for enzyme immobilization [3–9], drug delivery [10,11], energy-harvesting devices [12–17] and biosensing [18,19], due to its large internal surface area, prodigious pore volume, biodegradability, and tunable pore geometry via variation of both porosity and crystallite size [20–24]. The biodegradation of porous silicon results in the formation of orthosilicic acid, which can easily be absorbed from the gastrointestinal tract and excreted from the body [25]. In addition, porous silicon can easily be made with the biomolecules and used in imaging and tumour targeting, although the detection mechanism is based on variation in either the photoluminescence spectra or the diffraction patterns [26–28]. As a result of the outstanding biocompatibility of porous silicon, it is popular in the biotechnology field for catalytic functions and enzyme immobilization [29]. Enzyme immobilization on a solid host might be favoured over its free counterpart [30,31] because immobilized enzyme has several advantageous features as a result of its reusability, prolonged shelf-life, better thermal and storage stability, and ease of separation of the enzyme from the reaction mixture with no enzyme contamination of the product [32–56].

In the present study, physical adsorption methodology was used to immobilize acetylcholinesterase on porous silicon architecture and the hydrolytic response towards acetylthiocholine iodide was assessed using a spectrophotometric bioassay. The porous silicon-immobilized acetylcholinesterase generates synergistic effects, which provide convenient enzyme handling, dexterous segregation from the reaction mixture, easy recycling protocols and reusability of the biocatalyst. The photoluminescence properties of a mesoporous silicon surface before and after enzyme immobilization suggest that enzyme localized on the porous silicon surface shows a slight enhancement in the photoluminescence emission intensity of the bare porous silicon rather than destruction of the visible photoluminescence of porous silicon. The cathode luminescence (CL), alongside fluorescence excitation and emission spectra before and after enzyme immobilization, can be used as an efficient reporting platform for successful enzyme adsorption on to the porous silicon surfaces with retention of immobilized enzyme activity being easily assessed by spectrophotometric bioassay.

## EXPERIMENTAL

### Materials and instrumentation

During the experiment the following were used: acetylcholinesterase (acetylcholine hydrolase, EC 3.1.1.7, acetylcholinesterase from human erythrocytes), acetylthiocholine iodide, 5,5′-dithio-bis(2-nitrobenzoic acid), neostigmine methyl sulfate and MgCl_2_, purchased from Sigma-Aldrich, NaCl (Daejung Chemical and Metals Co., Ltd), ethanol, water (Samchun Chemicals), HF (48%, w/w; Merck) and boron-doped p-type silicon wafers with a resistivity of 1–20 Ω cm and thickness 500–550 μm (obtained from Cree Co.). The photoluminescence spectra and relative photoluminescence intensities were measured on an FS-2 fluorescence spectrometer (Scinco) and LabRam HR-800 spectrometer (Horiba Jobin Yvon) with a He/Cd laser source (325 nm). Enzyme immobilization on the surface of porous silicon was confirmed by photoluminescence measurement through study of the changes in the photoluminescence intensity of porous silicon before and after enzyme adsorption. Pore size and porous film thickness were determined by field emission (FE)-SEM images (MIRA3 LMH, TESCAN). Cross-sectional views were obtained by etching on the reverse of the slide and breaking the sample. The Fourier transform IR (FT-IR) spectra were recorded using a PerkinElmer FT-IR spectrometer connected to an Auto IMAGE FT-IR microscope. The elemental analysis was performed with energy-dispersive X-ray spectroscopy (EDS) analysis, using FE-SEM, and X-ray photoelectron spectroscopy (XPS) analysis, using a VG surface analysis system (Thermo VG Scientific, ESCA Lab. 2000).

### Enzyme activity measurements

The inhibitory activity of acetylcholinesterase (acetylcholinesterase from human erythrocytes, 0.03 units/ml) was determined spectrophotometrically using acetylthiocholine iodide as a substrate and the reported method of Ellman et al. [57]. Briefly, the assay solution consisted of 180 μl of 50 mM Tris/HCl buffer, pH 8.0, containing 0.1 M sodium chloride, 0.02 M magnesium chloride, 20 μl of enzyme and 15 μl of 14.9 μM neostigmine methyl sulfate, and pre-incubated for 30 min at 4°C. To this reaction mixture 5,5′-dithio-bis(2-nitrobenzoic acid) (0.3 mM, 20 μl) and acetylthiocholine iodide (1.8 mM, 20 μl) were added and incubated for 10 min at 37°C, followed by measurement of the absorbance at 412 nm. The same assay procedure was followed for immobilized enzyme. The assay measurements were carried out using a micro-plate reader (OptiMax, Tunable Micro plate Reader; wavelength range 340–850 nm for 96-well plates). The reaction rates of immobilized enzyme were compared with its free counterpart, and the percentage inhibition was calculated using the formula 100−(Abs_testwell_/Abs_control_)×100 [58], where Abs_testwell_ is the absorbance of the well being tested and Abs_control_ the absorbance of the control sample.

### Preparation of porous silicon surface

The silicon wafer (boron-doped p-type silicon wafers with resistivity 1–20 Ω cm and thickness 500–550 μm) was cut into chips sized 1×1 cm^2^ and degreased with an ultrasonic bath of acetone for 5 min, and then rinsed in deionized water. After drying with nitrogen gas, silver paste was simply sputtered on to the back of the wafer to provide a back ohmic contact for anodization. Adhesive tape (ASF-110) was used to bind wire and a 0.25-cm^2^ paper section was used on the front of the silicon wafer. The paper section was removed to expose 0.25 cm^2^ of the silicon wafer surface, which was then rinsed with trichloroethylene, acetone and deionized water. The porous layer was prepared on a p-type silicon wafer by using electrochemical anodization. The silicon wafer was anodized at a current density of 20 mA/cm^2^ using a Keithley 2400 source meter in HF (48%, w/w; Merck) electrolyte solution (HF:H_2_O:C_2_H_2_OH=1:1:2, by vol.) for 5, 10, 15, 20, 25, 30 and 35 min, respectively, in dark conditions to obtain an appropriately structured sample. The counter electrode used during the etching process was platinum. Immediately after anodization, the sample was taken out from the cell, rinsed in deionized water and left to dry in nitrogen gas.

### Immobilization of the enzyme on the porous silicon surface

A total of 20 μl (0.03 units/ml) of acetylcholinesterase was dropped on to the surface of the porous silicon and allowed to dry. The enzyme loading was optimized by using different volumes of enzyme in the range 10–30 μl on the porous silicon sample. Porous silicon samples obtained by varying the etching time from 5 min to 35 min, with constant current density, were tested for immobilization of acetylcholinesterase, and the efficiency of the immobilization was confirmed by studying the retention of activity on the surface of the porous silicon spectrophotometrically. However, the samples prepared with a current density of 20 mA/cm^2^ for 30 min showed optimum results and were therefore used for further measurements.

## RESULTS AND DISCUSSION

**Figure 1:**
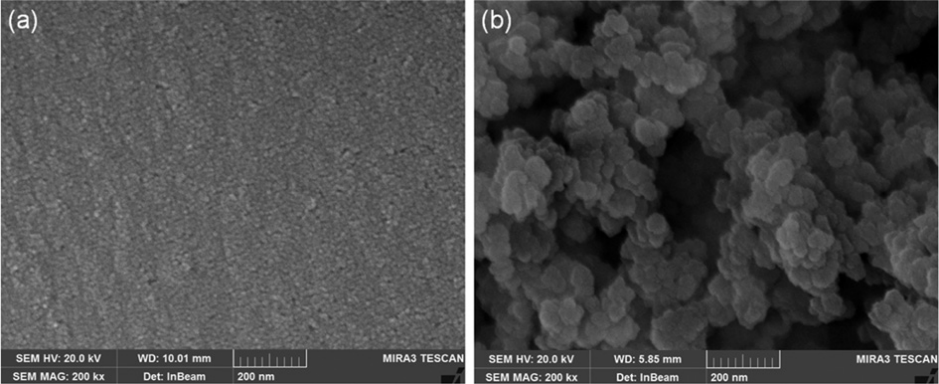
Plan-view FE-SEM Plan-view FE-SEM of (**a**) bare porous silicon surface and (**b**) acetylcholinesterase-immobilized porous silicon surface.

**Figure 2:**
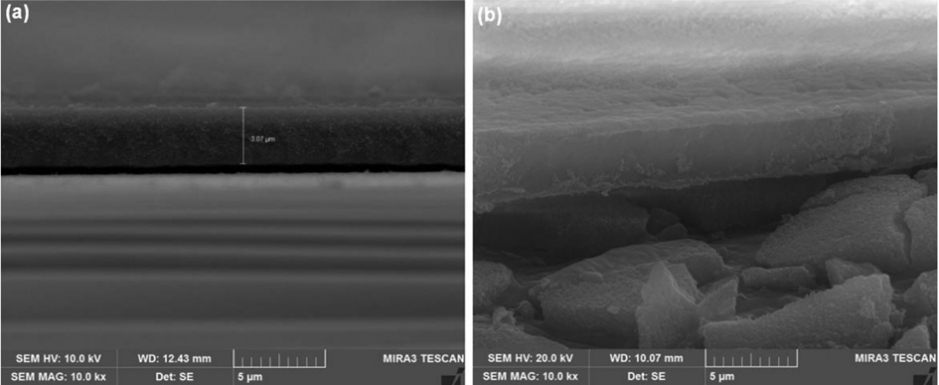
Cross-sectional FE-SEM view Cross- sectional FE-SEM of (**a**) bare porous silicon surface and (**b**) acetylcholinesterase-immobilized porous silicon surface.

To investigate the development of a nano-porous silicon layer during the etching process, the surface morphology was examined using FE-SEM analysis before and after enzyme immobilization. Figure 1(a) shows the nano-porous silicon surface before enzyme immobilization whereas the micrograph in Figure 1(b) shows the enzyme-immobilized porous silicon surface. It can be observed that the nano-porous silicon layer has a pore size in the range 2–50 nm (Figure 1a) and a porous film thickness of 3–4 μm (Figure 2a). Figures 1(b) and 2(b) represent the enzyme adsorption on to the porous silicon surface and the efficiency of hydrolysis of the immobilized enzyme was assessed using the spectrophotometric bioassay utilizing neostigmine methyl sulfate as a standard acetylcholinesterase inhibitor [59–61].

The cross-sectional FE-SEM images were obtained to discriminate the porous surface from the enzyme-immobilized porous surface. In the cross-sectional FE-SEM micrograph, the porous silicon layer appears to be similar to a sponge-like thin layer, and there is adsorption of enzyme on to that sponge-like porous surface through the creation of a second microlayer of enzyme (Figure 2). The enzyme was observed to be retained on the porous layer with no penetration into the bulk of the porous surface. The major trapping forces for a thin enzyme layer on the porous surface were considered to be van der Waals' forces and hydrogen bonding between the enzyme's hydroxyl group and the porous layer's hydrogen atoms.

### FT-IR analysis

**Figure 3:**
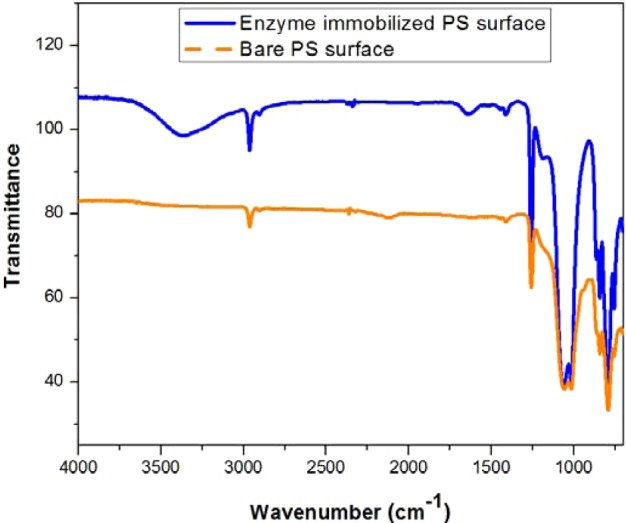
FT-IR spectra FT-IR spectra of bare porous silicon surface and enzyme-immobilized porous silicon surface.

The FT-IR analysis was done to determine the chemical composition of the samples. The presence of enzyme in the porous silicon material was indicated in the FT-IR spectra by the appearance of a new broad signal at 3364 cm^−1^, assigned to acetylcholinesterase's free hydroxyl stretching mode, whereas no such signal was detected in the highlighted area on the bare porous silicon spectrum. The sharp signal at 2962 cm^−1^ for both bare and enzyme-immobilized porous silicon surfaces was attributed to C–H stretching vibrations due to adsorbed hydrocarbons on the surface. The appearance of a sharp as well as a broad-band signal, with less growth in the intensity at the spectral span from 2335 cm^−1^ to 2119 cm^−1^, is characteristic of the Si–H bond, which partially vanished after acetylcholinesterase immobilization. The broad-band signal at 1641 cm^−1^ was also observed on the enzyme-immobilized surface associated with the NH bending and scissoring mode, although no such signal was harvested for bare porous silicon. Meanwhile, a sharp signal in the range 1451–1410 cm^−1^ was assigned to the C==C stretching vibration arising from the immobilized enzyme skeleton present on the porous surface (Figure 3).

### EDS analysis

**Figure 4:**
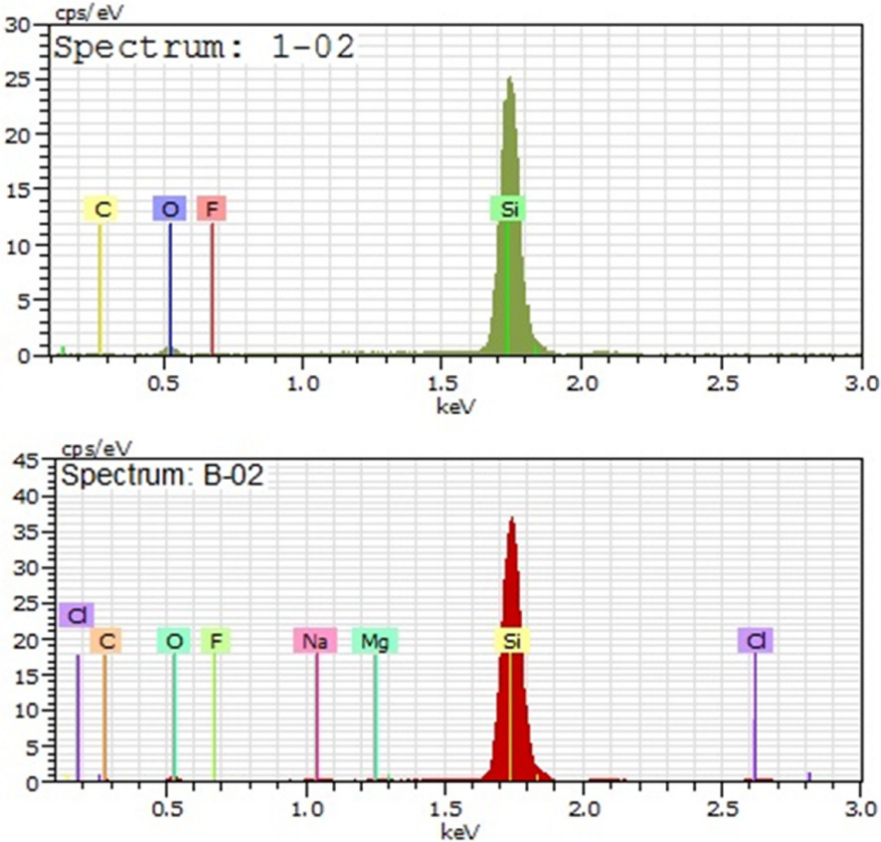
EDS data EDS data for bare porous silicon surface (spectrum: 1-02) and acetylcholinesterase-immobilized porous silicon surface (spectrum: B-02).

The elemental analyses of the bare porous silicon surface, as well as the acetylcholinesterase-immobilized porous silicon surface, were done to gain insight into the presence of enzymes on the porous silicon surface using EDS analysis. There was an appreciable appearance of new elements, including chlorine, magnesium and sodium, which are attributed to the enzyme skeleton adsorbed on to the porous silicon surface. Moreover, the weight percentage of silicon, fluorine and carbon considerably varied in the EDS spectrum after enzyme immobilization, giving further evidence of the presence of enzyme on the porous silicon surface, as shown in Figure 4.

### XPS analysis

**Figure 5:**
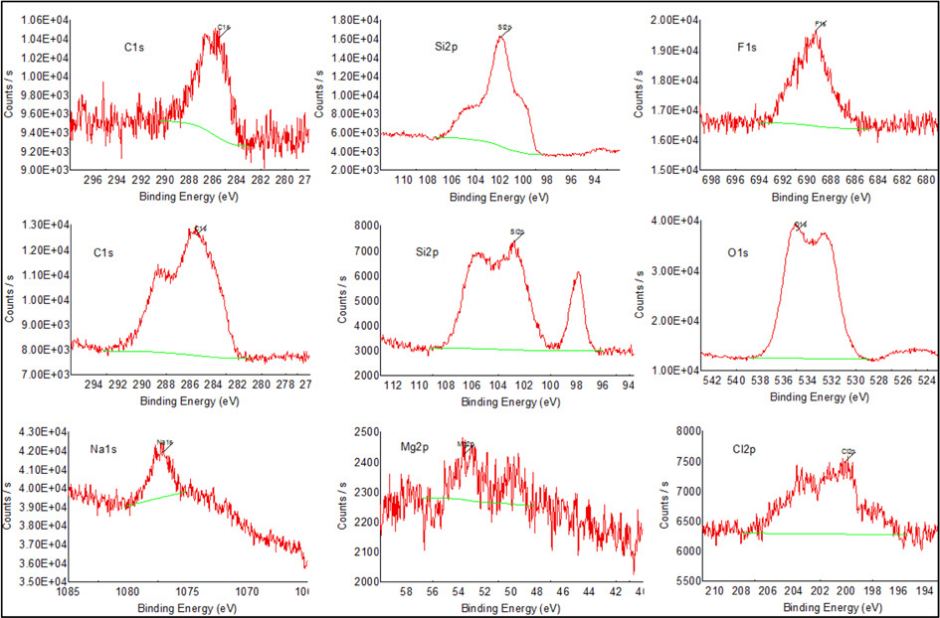
XPS analysis data XPS analysis for the bare porous silicon (first-row spectra) and acetylcholinesterase-immobilized porous silicon (second- and third-row spectra), along with their binding energy values–obtained for carbon, fluorine, oxygen and sodium at 1s core level, and silicon, magnesium and chlorine at 2p core level.

To diagnose the particular chemical species and the electronic environments at the surface, the XPS analysis was performed using VG surface analysis system (Thermo VG Scientific, ESCA Lab. 2000) before and after the enzyme was immobilized on the porous silicon surface. During XPS analysis, the sample was irradiated with monoenergetic X-rays, causing photoelectrons to be emitted from the sample surface. An electron energy analyser determines the binding energy of the photoelectrons and, from this and the intensities of the photoelectron peak, the element's identity, chemical state and quantity were determined. The information from XPS analysis was used for analysis of bare and acetylcholinesterase-immobilized surface layers. The original XPS spectrum obtained by analysing bare porous silicon and enzyme-immobilized porous silicon for several elements present on the surface is shown in Figure 5.

The results of XPS analysis are consistent with EDS analysis. The detected elements on the surface and their weight percentage for bare and immobilized porous silicon samples are shown in Table 1. There was a considerable increment in the weight percentage of carbon from 6.21% to 24.62% after enzyme immobilization, indicating the hydrocarbon structure of the enzyme trapped on the porous silicon, whereas there was a remarkable decline in the silicon weight percentage and complete elimination of fluorine on the enzyme-immobilized porous silicon, indicating the surface compensation by enzyme. Meanwhile, the expected high weight percentage of oxygen due to acetylcholinesterase's free hydroxyl group and manifestation of extra elements, including magnesium, chlorine, sodium and nitrogen, was expected from the adsorbed acetylcholinesterase skeleton.

**Table 1:** XPS data with corresponding weight percentage of chemical species perceived on the porous silicon surface before and after enzyme immobilization

	Bare porous silicon		Enzyme-immobilized porous silicon	
Species no.	Elements	Weight percentage	Elements	Weight percentage
1	C	6.21	C	24.62
2	F	4.86	–	–
3	Si	88.94	Si	27.19
4	–	–	Na	0.69
5	–	–	O	41.78
6	–	–	Mg	1.17
7	–	–	Cl	2.61
8	–	–	N	1.94

### Photoluminescence and CL measurements

**Figure 6:**
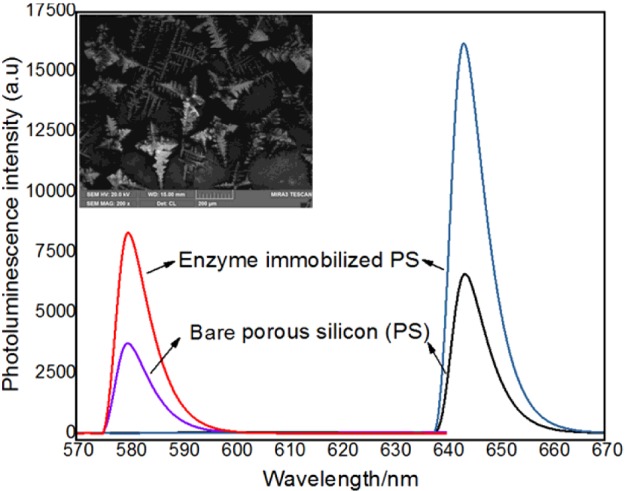
Photoluminescence excitation spectra Photoluminescence excitation spectra (left side spectra) of bare porous silicon, as well as enzyme-immobilized porous silicon surface, and photoluminescence emission spectra (right side spectra) of bare and acetylcholinesterase-immobilized porous silicon surface, whereas a CL spectrum (inset) was obtained to get further assistance in understanding the photoluminescence enhancement from a porous silicon surface after enzyme immobilization (the scale bar for the inset is 200 μm); a.u., absorbance units.

The porous silicon surface displayed variable dynamic features in the tuning surface chemistry, especially, in the case of photoluminescence on porous silicon, where the surface adsorbent may just block the photoluminescence process or cause a retardation of electronic excitation; however, some chemical species may act as electron donors or acceptors, thus leading to quenching or enhancement of surface luminescence [62]. In the present study, the photoinduced visible light emission from lightly oxidized porous silicon showed photoluminescence emission maxima at 643 nm and fluorescence excitation maxima at 579 nm. There was a sudden increment in the intensity of porous silicon excitation and emission spectra after enzyme immobilization. The enzyme was trapped on the porous silicon, with no destruction of its photoluminescence and slight enhancement in the photoluminescence intensity, whereas free enzyme in solution did not exhibit any photoluminescence. However, following enzyme immobilization on porous silicon architecture, the enzyme aggregates on porous silicon were found via luminescence, assessed by photoluminescence as well as CL using a fluorescence spectrometer and FE-SEM, respectively. In the CL spectrum, the brightness from the area of enzyme aggregation (inset in Figure 6) is due to luminescence from the immobilized enzyme on the porous silicon surface. These CL spectra, alongside photoluminescence excitation and emission spectra before and after enzyme immobilization, can be used as an efficient reporting platform for successful enzyme adsorption on to the porous silicon surface. Moreover, the immobilized enzyme is retained on the porous silicon surface without obliteration of its luminescence behaviour at room temperature, as shown in Figure 6.

### Acetylcholinesterase inhibition assay for immobilized and free enzyme

**Figure 7:**
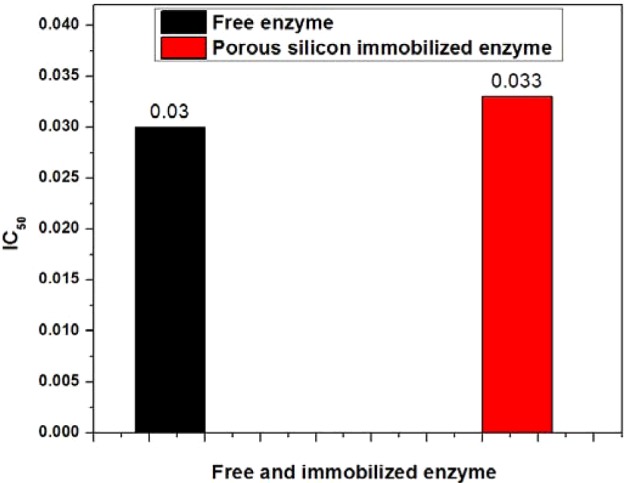
Comparison of IC_50_ values Comparison of IC_50_ (μg/ml) values for free and porous silicon-immobilized acetylcholinesterase.

To assess the hydrolysis response of acetylcholinesterase towards acetylthiocholine iodide, the spectrophotometric bioassay was performed using the standard acetylcholinesterase inhibitor (neostigmine methyl sulfate, 14.9 μM). For a comparative study, both free and porous silicon-immobilized enzymes were exploited in the spectrophotometric bioassay. The 50% enzyme inhibition was observed at 0.03 μg/ml of neostigmine methyl sulfate for the free enzyme, whereas porous silicon-immobilized acetylcholinesterase exhibited 50% activity at a drug concentration of 0.033 μg/ml, which was almost the same as that of the soluble enzyme. An activity close to that of the porous silicon-immobilized enzyme, with its free counterpart, indicates successful enzyme adsorption on to the porous silicon surface, alongside retention of its hydrolysis response towards acetylthiocholine iodide, as shown in Figure 7.

### Reusability

**Figure 8:**
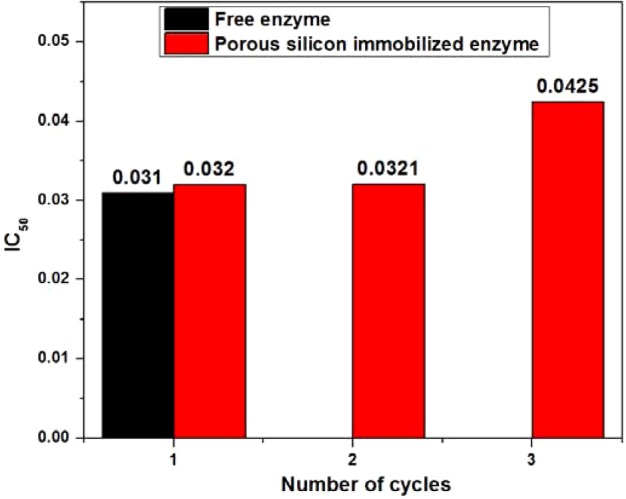
The IC_50_ values The IC_50_ (μg/ml) values of free enzyme and porous silicon-immobilized acetylcholinesterase after repeated use for up to three cycles.

One of the most important properties of the immobilized enzyme, primarily due to its reusability, is the prerequisite that commercialization of biocatalysts demands that the enzymatic process be economically cost saving; this makes it superior to the free counterpart by assembling an enzymatic process that is economically favourable and deeply rooted in the field of biotechnology [63,64]. To find out how reusable the enzyme is, the spectrophotometric assay was conducted using varying concentrations of neostigmine methyl sulfate and half-maximal inhibitory concentration (IC_50_) values were calculated to achieve 50% enzyme inhibition with the drugs used. From Figure 8, it is clear that, for the immobilized enzyme, the enzyme inhibition activity was quite similar, compared with the free enzyme, for up to three batches. However, during the third cycle, a slight reduction in immobilized enzyme activity was observed, because 50% enzyme activity was obtained at a slightly higher drug consumption (0.04 μg/ml of neostigmine methyl sulfate) compared with the first and second cycles. However, at the fourth cycle of reusability, the enzyme activity was reduced by up to 36% and the IC_50_ values could not be calculated. From these results, it was concluded that porous silicon-immobilized enzyme can be used for up to three cycles, with a promising hydrolytic response towards acetylthiocholine iodide, although afterwards a gradual decrease in enzyme activity would make it unfeasible to obtain 50% enzyme inhibition with the standard treatment drug. However, the immobilized enzyme is completely inactivated after five batches.

### Storage stability

**Figure 9:**
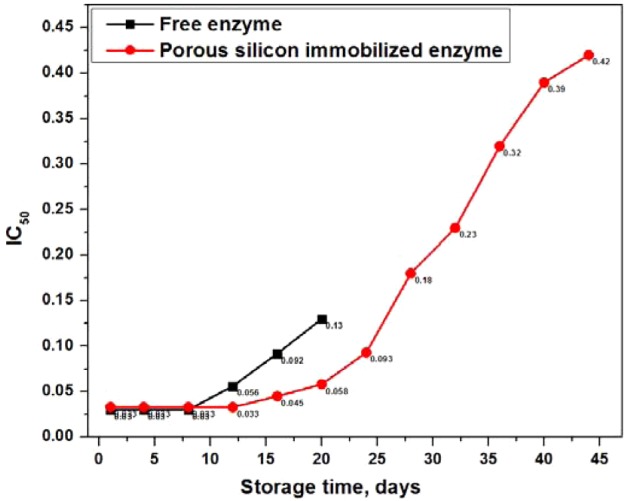
Comparison of IC_50_ values Comparison of IC_50_ (μg/ml) values of free enzyme with those of porous silicon-immobilized acetylcholinesterase, with the passage of time, to assess storage stability by keeping both of them at 4°C, followed by continuous measurement of their activity at regular 4-day intervals.

To determine the storage stability of porous silicon-immobilized acetylcholinesterase, the immobilized enzyme, as well as its free counterpart, were stored at 4°C and their hydrolytic response towards acetylthiocholine iodide was evaluated after regular 4-day intervals by spectrophotometric bioassay. The free enzyme showed very similar activity, as well as the porous silicon-immobilized acetylcholinesterase, for up to 8 days; there was then a continuous decrease in the activity of the free enzyme and it was completely inactivated after 20 days. However, the immobilized enzyme retained 50% activity, comparable to that of free enzyme over a longer period of time, and preserved its 50% activity at slightly higher drug concentrations for up to 44 days. Thereafter, the activity of the immobilized enzyme was reduced below 50%, as shown in Figure 9. These results lead us to conclude that the immobilized enzyme possesses significant advantages over the free enzyme because of its longer shelf-life, improved stability, convenient handling, inactivity when separated from the reaction mixture and prevention of contamination by the enzyme's products.

### Thermal stability

**Figure 10:**
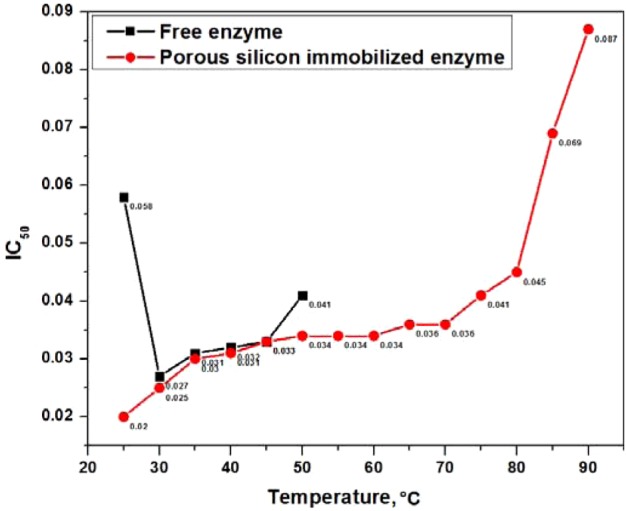
Thermal stabilities Thermal stability of free enzyme and porous silicon-immobilized enzyme by incubating both at a variable temperature for 1 h.

To investigate the effect of temperature on the inhibition potential of immobilized enzyme towards neostigmine methyl sulfate, the free and porous silicon-immobilized enzyme were incubated at different temperatures ranging from 20°C to 120°C for 1 h, followed by measurement of the enzyme activity spectrophotometrically. The IC_50_ values for free and immobilized enzyme were calculated using different concentrations of neostigmine methyl sulfate, ranging from 0.0091 μg/ml to 0.294 μg/ml. The free acetylcholinesterase exhibited 50% activity with varying concentrations of neostigmine methyl sulfate up to 50°C; latterly there was complete inactivation of the free enzyme. Meanwhile, the porous silicon-immobilized enzyme displayed 50% inhibitory activity over a bigger temperature span, up to 90°C, as shown in Figure 10. The immobilized enzyme resists thermal denaturation over much higher temperatures compared with the free enzyme, and this interesting operational feature of immobilized enzyme under harsh reaction conditions could contribute to the enzyme's longer shelf-life.

### The pH effect

**Figure 11:**
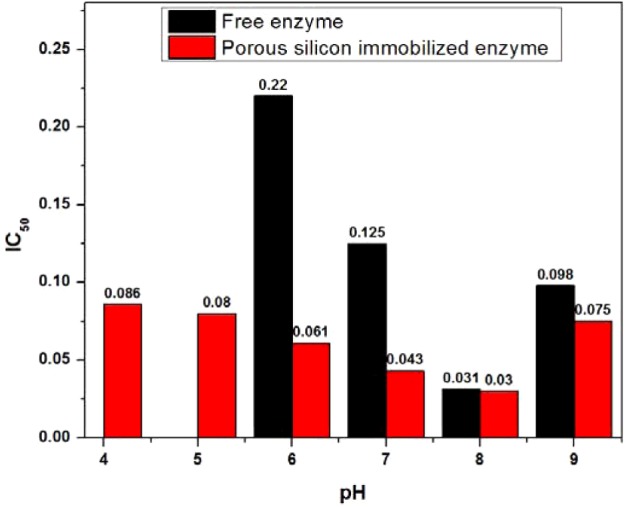
The pH stability The pH stability of free and immobilized enzyme for assessment of the optimal pH for enzymatic activity.

The effect of pH was evaluated in the pH range 4–9 to figure out the optimal pH span for the immobilized enzyme. The results showed that the enzyme's activity strongly depended on the buffer's pH. The free and immobilized enzymes work well at pH 8, but there was less than 50% enzyme activity left at pH <6, as well as pH >9 for the free enzyme, whereas the immobilized enzyme retained 50% activity over a broad pH span–4–9. In the meantime, 50% enzyme activity was obtained with slightly larger amounts of drug for the free enzyme at a pH below and above 8, whereas the immobilized enzyme remained stable, with a better activity in the pH range shown in Figure 11. This property of the immobilized enzyme makes it a reliable source for enzyme catalysis reactions over a broad pH spectrum.

## CONCLUSION

Acetylcholinesterase has been successfully immobilized on the nano-porous silicon surface; the porous surface suitable for enzyme immobilization was found by changing the etching parameters. The hydrolytic response of the immobilized enzyme towards acetylthiocholine iodide was assessed using a spectrophotometric bioassay. The percentage inhibitions of the immobilized as well as the free enzyme-catalysed reactions were monitored using the standard acetylcholinesterase inhibitor neostigmine methyl sulfate. The immobilization processes considerably enhance storage stability for up to 44 days, with retention of 50% enzymatic activity and application of the enzyme in slightly harsh conditions (thermal stability up to 90°C), easy recovery from the media with no contamination of the reaction mixture, reusability for up to three cycles and pH stability over the broad span 4–9. This finding suggests that the immobilized enzyme can be capitalized on as a most promising technique in organic and biomolecule synthesis, with satisfactory technological and economical advantages over the free enzyme due to its recycling, ease of separation from the reaction mixture and superficial handling strategy. The simple adopted immobilization procedure provides very good enzyme adsorption on to the nano-channel of a mesoporous silicon surface, with minimal leaching and an abundant hydrolytic response towards acetylthiocholine iodide, comparable with soluble enzyme, while the enzyme adsorption on to the porous silicon architecture can easily be inspected using photoluminescence measurements, spectrophotometric assay, FE-SEM, FT-IR, EDS analysis, CL measurement and XPS analysis.

## Abbreviations

CL: cathode luminescence
EDS: energy-dispersive X-ray spectroscopy
FE-SEM: field emission SEM
FT-IR: Fourier transform IR spectroscopy
XPS: X-ray photoelectron spectroscopy
